# Designing and Conducting Motivational Interviewing Research in Veterinary Clinical Settings: A Practical Guide for Researchers

**DOI:** 10.3390/ani16132077

**Published:** 2026-07-05

**Authors:** M. Carolyn Gates, Clare J. Phythian, Eileen Britt

**Affiliations:** 1 AkoVet Limited, Palmerston North 4410, New Zealand; 2 School of Psychology, Speech, and Hearing, University of Canterbury, Christchurch 8140, New Zealand; eileen.britt@canterbury.ac.nz

**Keywords:** motivational interviewing, research methodology, fidelity, study design, clinical settings, outcome measurement, veterinary medicine, implementation, behaviour change

## Abstract

Motivational interviewing (MI) is gaining traction in veterinary medicine as an evidence-based approach for engaging clients in conversations that strengthen their intrinsic motivation to change behaviours that affect the welfare of animals in their care. While MI has a substantial and well-established evidence base across human healthcare and related fields, the veterinary evidence base remains small and is currently limited by study design challenges, inconsistent fidelity measurement, and outcome measures that rarely extend beyond short-term changes in communication behaviour. Existing methodological guidance addresses treatment integrity and theoretical mechanisms but does not provide an integrated framework for designing robust studies across the range of questions relevant to veterinary practice. This paper addresses that gap by providing practical methodological guidance for researchers designing MI studies in veterinary clinical settings. A novel conceptual model of the MI implementation pathway, mapping the journey from practitioner awareness through training and delivery to client outcomes, is used to organise key research questions that need to be answered in veterinary contexts. Guidance is then provided on selecting appropriate study designs, measuring fidelity and outcomes, estimating sample size, and managing the practical and ethical challenges specific to veterinary clinical settings, alongside minimum reporting standards to support more rigorous and reproducible MI research.

## Simple Summary

The way in which veterinarians communicate with clients can significantly influence clinical outcomes. Motivational interviewing is a communication approach that helps people find their own reasons for making changes, rather than being told what to do, and has shown promise in veterinary medicine. However, the research conducted so far has been limited by study design challenges, inconsistent measurement of whether motivational interviewing was delivered properly, and outcome measures that rarely capture whether consultations led to real improvements in animal welfare. This paper provides practical guidance for researchers wanting to design better studies on motivational interviewing in veterinary settings. A conceptual model maps the journey from a veterinarian first learning about motivational interviewing through to delivering it skillfully in clinical practice and is used to identify the most important research questions that still need to be answered. The paper then offers guidance on choosing appropriate study designs, measuring whether motivational interviewing was delivered correctly, estimating how many participants are needed, and reporting results in enough detail for others to understand and build on the work. The goal is to help build a stronger and more useful evidence base for motivational interviewing in veterinary practice.

## 1. Introduction

Motivational interviewing (MI) is an evidence-based communication approach that has been widely adopted across human healthcare to help people explore their own reasons for change and build the intrinsic motivation needed to act on clinical recommendations [1,2]. Its application to veterinary medicine reflects growing recognition that how veterinarians communicate with clients during a consultation, and not just what they advise, also plays a critical role in whether recommendations translate into sustained behaviour change and improved animal welfare [3,4,5]. Previous research into veterinary communication has consistently found that veterinarians have a predominantly directive and paternalistic consultation style that may actively undermine the outcomes it seeks to produce [6,7,8,9], since directive persuasive communication is more likely to generate resistance than to build the internal motivation that produces sustained behaviour change [10,11,12]. MI provides veterinarians with a framework that shifts the focus of clinical conversations from information delivery and persuasion to eliciting the client’s own motivation for change.

A companion narrative review to this paper evaluated the current veterinary MI literature and found that while the available studies are broadly supportive of MI as an approach, the evidence base remains small and is currently limited by study design challenges, inconsistent fidelity measurement, and outcome measures that rarely extend beyond short-term changes in communication behaviour to capture downstream effects on client behaviour or animal welfare [13]. This pattern is not unique to veterinary medicine. Systematic reviews and meta-analyses of MI conducted across human healthcare settings have repeatedly found that the evidence base is undermined by studies that are either too poorly designed to support robust conclusions or described in insufficient detail to allow meaningful comparison across the literature [14,15,16,17]. Inadequate reporting of training, absent or inconsistent fidelity assessment, and heterogeneous outcome measurement have been identified as primary limiting factors across multiple independent reviews of the MI literature in primary care and medical settings making it difficult to determine what was actually delivered in many studies, whether it constituted true MI, and whether any effects observed can be attributed to the intervention itself or to variation in how it was implemented [14,15,16,17].

Veterinary clinical practice introduces additional structural challenges that are important to factor into research study designs. In contrast to the addiction counselling and mental health settings where MI was originally developed, medical and veterinary consultations are typically short, time-pressured, and often span multiple clinical problems, which limits the opportunities that veterinarians have to develop their MI skills and may not generate sufficient behaviour change focused content to support accurate fidelity coding. The triadic nature of veterinary consultations, where the veterinarian and client share decision-making authority over an animal who cannot participate in the conversation, creates motivational and ethical dynamics that are less commonly encountered in the human healthcare settings where MI was developed and most extensively studied [13]. Furthermore, clients in veterinary consultations are being asked to change their own behaviour for benefits experienced primarily by their animal, which means the intrinsic motivation that MI is designed to evoke cannot be assumed to operate in the same way as most medical consultations [7]. This means that researchers cannot simply borrow study designs, fidelity tools, and outcome measures from human healthcare without carefully examining whether their underlying assumptions hold in a veterinary context.

A further challenge common across all areas of MI research is establishing whether practitioners reached sufficient MI proficiency through training and whether they maintained that proficiency when delivering MI in real clinical settings. Brief training consistently produces short-term improvements in confidence and skills but rarely translates into sustained changes in clinical behaviour [18,19]. Many studies also rely on self-reported fidelity assessment given the labour-intensive nature of recording and coding real consultations, which is problematic because practitioners systematically overestimate their own MI skills when assessed against independently coded recordings [20,21]. This means that end of training assessments and practitioner self-reports are unreliable indicators of whether MI is actually being delivered in practice, which has direct implications for how MI research studies are designed and what conclusions can be drawn from them [13]. With the veterinary MI evidence base still in its infancy, there is significant opportunity to build it on stronger methodological foundations than have characterised the human healthcare literature.

This paper addresses these challenges by providing a methodological guide for researchers designing MI studies in veterinary clinical settings. Following a brief introduction to MI and how it works in clinical practice, a novel conceptual model of the MI implementation pathway is introduced that maps the specific stages through which MI moves from practitioner awareness to skilled clinical delivery and meaningful client and animal outcomes. This model integrates established implementation science stages with MI-specific constructs, including the distinction between competency and proficiency and the proximal-to-distal chain from change talk to behaviour change to animal welfare, and is used here to organise the key research questions that need to be answered in veterinary contexts and to structure the guidance that follows. The paper then covers study design, outcome and fidelity measurement, sample size estimation, recruitment and retention, ethical considerations, and reporting standards, with the goal of supporting a more rigorous and comprehensive evidence base as the field continues to grow.

## 2. Methods

This paper is a methodological guidance document rather than a traditional systematic or narrative review of a defined evidence base because there are currently no peer-reviewed published guidelines for designing and conducting MI research in clinical settings. The impetus for this paper came from a pattern identified across systematic reviews and meta-analyses of the MI literature in human healthcare, which repeatedly flagged poor study design, inconsistent fidelity measurement, and inadequate The guidance presented draws on three sources: the methodological recommendations and identified gaps in systematic reviews, the authors’ combined experience designing and evaluating research studies across multiple settings, and a companion narrative review of the veterinary MI evidence base that documented the same limitations in the smaller veterinary literature [13].

Supporting literature was identified through searches of PubMed, Web of Science, and Google Scholar using the terms ‘motivational interviewing,’ ‘fidelity assessment,’ ‘study design,’ ‘behaviour change,’ ‘implementation,’ ‘training,’ and ‘outcome measurement,’ used individually and in combination, supplemented by hand-searching of reference lists and direct consultation of foundational MI methodology resources including published coding manuals. Sources were selected on the basis of relevance to the methodological guidance being developed rather than to characterise the breadth of a defined literature.

## 3. Understanding MI and How It Works in Practice

Developing robust MI research studies requires a good understanding of what MI is, how it is structured and delivered in clinical practice, and how fidelity is assessed. This section covers the core principles, skills, tasks, and fidelity assessment tools that underpin MI use in veterinary clinical settings before describing the implementation pathway from initial engagement through training and sustained delivery, all of which provide essential background context for the methodological guidance that follows.

### 3.1. MI in Practice: Principles, Skills, and Veterinary Considerations

The fundamental premise of MI is that people are more likely to implement sustainable behaviour change when they verbally articulate their own reasons for change and develop an action plan that aligns with their lifestyle, preferences, and goals [22]. Rather than persuading or directing the client, the MI practitioner’s role is to listen carefully to what the client is saying and strategically use core communication skills (open-ended questions, affirmations, reflections, and summaries, collectively known as OARS) to strengthen change talk, soften sustain talk, and guide the conversation toward a mutually agreed behavioural target [1]. Change talk refers to any client speech that favours movement toward change, including statements about desire, ability, reasons, need, and commitment to change while sustain talk refers to speech that favours the status quo, including arguments against change or in favour of continuing current behaviour.

In practice, MI conversations are structured around four sequential tasks: engaging, focusing, evoking, and planning. Engaging establishes a working relationship and creates a safe environment in which the client feels heard and understood. Focusing identifies and clarifies the specific behaviour change that will be the target of the conversation. Evoking draws out the client’s own motivations, values, and reasons for change, and is where the core work of MI occurs. Planning consolidates commitment and supports the client in developing a concrete, achievable plan of action. While these tasks are broadly sequential, skilled practitioners move fluidly between them in response to where the client is in the conversation, and not every consultation will progress through all four.

Underpinning both components is the spirit of MI, which is how the practitioner should be experienced as working, operationalised through the PACE principles: partnership, acceptance, compassion, and empowerment [1]. Partnership reflects the collaborative nature of MI, where the practitioner and client work together rather than the practitioner assuming an expert role and directing the conversation toward a predetermined outcome. Acceptance acknowledging the client’s perspective without judgment, even when their current behaviour is causing harm. Compassion reflects a commitment to the client’s wellbeing and best interests, ensuring that the practitioner’s motivations remain oriented toward helping rather than persuading. Empowerment recognises the client’s autonomy, seeks to draw on their strengths, and recognises that the capacity and motivation for change lie within the client and that the practitioner’s role is to draw these out rather than impose them from outside. These principles ensure that MI remains a collaborative process oriented toward the client’s own values and goals, rather than a persuasion technique directed toward outcomes the practitioner considers desirable.

Clinical consultations often require the practitioner to share clinical findings, explain diagnoses, and make recommendations for treatments. This is not incompatible with MI but requires careful delivery. Providing unsolicited information or advice in a directive way is one of the most common MI-non-adherent behaviours in clinical practice that can push clients further away from change. The Ask–Provide–Ask framework offers a practical structure for sharing information in an MI-consistent manner. The practitioner first asks what the client already knows or understands about the issue and then seeks permission to share additional information that the client needs to make a decision. The information or recommendations are then provided clearly and non-prescriptively, ideally tailored towards the client’s own situation. The practitioner then asks how the client has received the information, what questions they have, and what it means for them, creating space for the client to respond, raise concerns, or express ambivalence. Used well, Ask–Provide–Ask transforms the information-giving component of a consultation from a one-way transfer of expertise into a collaborative exchange that keeps the client’s autonomy and perspective at the centre of the conversation.

Delivering MI well requires attention to two distinct components: relational and technical. The relational component refers to how well the client feels heard, understood, and able to trust the practitioner, and is foundational to creating the conditions in which behaviour change conversations can occur. The technical component refers to how well the practitioner guides the conversation to evoke and strengthen change talk and soften sustain talk. The primary tool used assess whether these components are being delivered as intended across MI research contexts is the Motivational Interviewing Treatment Integrity coding system (MITI 4.2.1) [23], which evaluates global dimensions of practitioner communication including empathy, partnership, and cultivating and softening change talk, alongside frequency counts of specific practitioner verbal behaviours across a coded segment of consultation audio. MITI-derived thresholds for fair and good competency provide benchmarks for evaluating whether training has been sufficient to produce clinically meaningful communication change. However, these thresholds were developed through expert consensus rather than empirical validation, and there is evidence that meaningful client change talk and behaviour change can occur even when practitioners fall below them, raising questions about whether they represent a universally appropriate benchmark for assessing clinical effectiveness [24,25].

Knowing when to use full MI, when to draw selectively on MI-consistent skills, and when the clinical situation calls for a different approach entirely is itself a marker of practitioner expertise that research designs need to account for. Treating MI as a binary intervention that is either delivered or not delivered misses the reality that skilled practitioners calibrate their approach to each consultation, and fidelity tools and outcome measures need to be sensitive enough to capture this variation rather than reducing it to a single pass or fail judgement.

### 3.2. From Engagement to Outcomes: The MI Implementation Pathway

**Figure 1:**
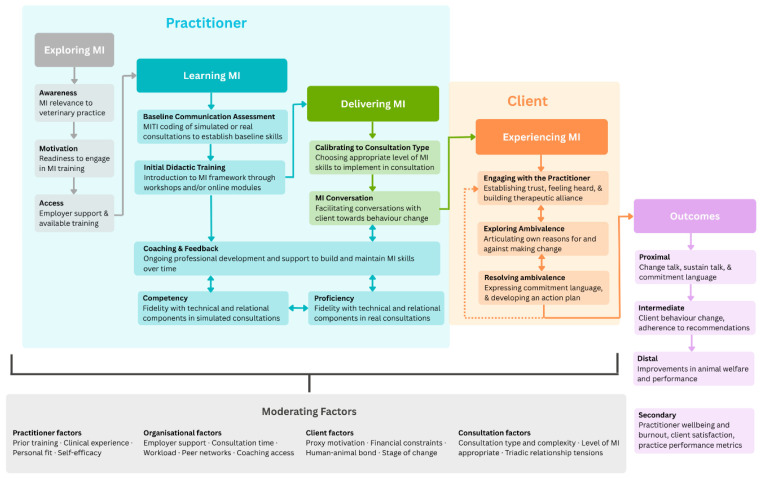
Conceptual model of the MI implementation pathway in veterinary clinical settings, showing the progression from practitioner exploration of MI through training, delivery, and client experience to proximal, intermediate, and distal outcomes. Arrows indicate the typical sequence of stages; feedback loops reflect the ongoing monitoring and skill maintenance required to sustain fidelity over time.

Implementing MI effectively in veterinary clinical settings requires practitioners to gain awareness of MI as an alternative approach to clinical conversations and then work through a systematic process of training, supervised practice, and ongoing skill development to reach and maintain the level of delivery needed to produce positive behavioural changes and better outcomes for animals and their owners. Most published MI research to date is geared towards understanding the different components of this process and how they can be influenced to increase the uptake and effectiveness of MI in clinical practice. Figure 1 provides an overview of MI implementation pathway, from initial exploration through training, delivery, and outcomes, and serves as the organising framework for the methodological guidance that follows.

Although the implementation pathway shown in Figure 1 presents MI adoption as a structured progression, in practice the pace and form of each stage varies considerably across practitioners and settings. Researchers have identified broad stages moving from exploration through adoption, initial implementation, and full implementation [26], but the decision to adopt MI and the speed at which practitioners move through each stage is shaped by a range of individual and organisational factors, including attitudes toward evidence-based practice, organisational climate, workload, and access to training and coaching resources [27]. Once MI is being delivered in practice, ongoing monitoring of consultation quality is important for maintaining fidelity over time. Without structured feedback, practitioners tend to drift from MI-consistent behaviours even after reaching competency thresholds.

The implementation pathway typically begins with exploration, where a practitioner or organisation becomes aware of MI and starts to assess whether it is relevant and achievable in their context. The decision to invest in MI training requires a degree of organisational readiness that is not always present. Consultation time is constrained, practice economics are tight, and adding structured communication training to an already demanding workload requires buy-in from employers and colleagues as well as from the practitioner themselves. Somewhat ironically, the process of getting practitioners on board with MI training often requires many of the same skills that MI itself aims to develop, including exploring ambivalence, eliciting intrinsic motivation, and supporting autonomous decision-making rather than directing.

Once the decision to proceed has been made, implementation moves into the process of learning MI. Initial training typically involves workshops or online modules that introduce the conceptual framework of MI alongside foundational communication skills. While this continues to build awareness and enthusiasm, it is rarely sufficient on its own to produce meaningful change in how practitioners communicate with clients. Consolidating skills to a level where they transfer reliably into real clinical practice requires sustained coaching and feedback, using both simulated and real consultation recordings as the basis for structured reflection. This is where the competency–proficiency distinction becomes practically important. Competency refers to the ability to demonstrate MI-consistent skills in controlled or simulated settings, while proficiency refers to consistent application under the conditions of real clinical practice, with all the time pressure, emotional complexity, and relational history that entails [22]. The two do not always develop in parallel, and a practitioner who performs well in a role-play scenario may still struggle to apply the same skills when a client is distressed, the appointment is running late, or the clinical picture is more complicated than expected. Even where practitioners do reach adequate proficiency, skills tend to decay without continued practice and structured feedback, and this decay can occur relatively quickly after training ends [18].

Delivering MI in practice also requires practitioners to make moment-to-moment decisions about when full MI is appropriate, when a lighter MI-informed approach better fits the consultation, and when the clinical situation calls for something else entirely. Not every consultation requires working through all four MI processes. Many will involve brief, targeted use of OARS skills or Ask–Provide–Ask within an otherwise standard clinical interaction. Skilled practitioners calibrate the level of MI they bring to each consultation based on client readiness, presenting problem, and available time, and research designs need to reflect this reality rather than treating MI as a uniform intervention that is either delivered or not.

While the practitioner is navigating these decisions, the client is having their own experience of the consultation. How a client experiences an MI-informed conversation, whether they feel heard, understood, and supported rather than directed or judged, shapes both their engagement during the consultation and their willingness to act on what was discussed. Clients who experience partnership and autonomy are more likely to articulate their own reasons for change, which in turn predicts subsequent behaviour change. This client-side experience is often overlooked in MI research, which tends to focus on practitioner behaviour and communication outcomes, yet it represents a critical link in the causal chain from MI delivery to real-world behaviour change.

Proximal outcomes such as the ratio of change talk to sustain talk within consultations provide an early indicator of whether MI is producing the conversational conditions associated with behaviour change, while intermediate outcomes such as client adherence and distal outcomes such as sustained behaviour change and improvements in animal welfare and production reflect the longer-term impacts of implementation. Secondary outcomes including practitioner wellbeing, job satisfaction, client satisfaction, and practice-level metrics such as consultation length and client retention are also relevant, since MI training has been associated with improved clinician wellbeing and reduced moral distress in human healthcare settings [28,29], and these effects may be particularly meaningful in the veterinary profession with its high rates of burnout and compassion fatigue [30,31]. Without tracking outcomes across these levels, it is difficult to determine where in the pathway a breakdown has occurred when MI does not produce the expected results.

This outcome stage of the implementation pathway is where the veterinary evidence base is weakest. Most existing studies have focused on proximal communication outcomes, particularly changes in practitioner behaviour following training, and very few have followed clients long enough to assess whether consultations that produced change talk translated into sustained behaviour change or measurable improvements in animal welfare. Outcome measurement in veterinary MI research also faces the additional complexity that the most meaningful outcomes, those experienced by the animal, are one step further removed from the intervention than in human healthcare, requiring researchers to trace a causal pathway from practitioner communication through client motivation through client behaviour through animal welfare, with each link introducing additional variability and attrition that makes effects harder to detect.

## 4. Generating MI Research Questions

Conducting robust MI research studies in veterinary clinical settings starts by asking the right research questions. The mechanisms through which MI produces behaviour change, including the role of change talk, the therapeutic alliance, and the balance between relational and technical components are well established in the broader MI literature [22] and are unlikely to be a primary research priority in veterinary contexts. However, questions still remain about whether these mechanisms operate in the same way when the client is a proxy decision-maker acting on behalf of an animal rather than for their own direct benefit and how to navigate difficult conversations where the practitioner needs to balance the interests of the animal against the autonomy of the client.

Table 1 maps key research questions across each stage of the MI implementation pathway based on the principle that MI research in veterinary contexts should be purposefully targeted towards improving the uptake and efficacy of MI in clinical practice. The list of questions presented in the table is not exhaustive and it should be noted that many studies will address questions that span multiple stages. For example, a study on training veterinarians in MI may touch on both Learning MI and Delivering MI if it assesses whether skills acquired in training transfer to real consultations, while an implementation study may encompass the full pathway from organisational readiness through to client outcomes.

Several frameworks have been developed to support the process of turning general research ideas like the ones presented in Table 1 into specific, testable questions to drive study design. Choosing the right one depends on the nature of the question being asked.

For exploratory or descriptive studies where there is no defined intervention, the Four Questions framework provides a flexible starting point. It asks (1) what the problem or issue of interest is, (2) who or what is affected, (3) what is being compared, changed, or explored, and (4) what outcome or insight are you trying to generate. This framework is particularly useful early in a research program when the study design is not yet fixed, or for descriptive studies characterising baseline communication in veterinary practice. An example would be: what gaps exist between how companion animal veterinarians in first-opinion small animal practice currently communicate with dog owners about weight management and MI-consistent communication standards to identify priorities for training program design? Here the problem is the gap between current practice and MI-consistent standards, the affected population is companion animal veterinarians and their dog-owning clients, what is being explored is the nature and extent of that gap, and the insight sought is identification of training priorities.

For questions evaluating the effect of an intervention, the PICOT framework provides the most widely used structure, specifying the Population being studied, the Intervention of interest, the Comparator against which it is evaluated, the Outcome being measured, and the Timeframe over which outcomes are assessed. PICOT is best suited to efficacy and effectiveness questions and training evaluation studies where a defined comparator condition exists. An example in veterinary MI research would be: in companion animal veterinarians with no prior MI training (P), does a six-month blended MI training program (I) compared with a single one-day workshop (C) increase the proportion reaching moderate MITI competency in real consultations (O) at twelve months post training (T)?

For questions focused on experiences, perceptions, or decision-making about MI rather than intervention effects, the SPIDER framework is more appropriate, specifying the Sample, the Phenomenon of Interest, the Design, the Evaluation, and the Research type. SPIDER suits qualitative and mixed-methods studies, including research into how practitioners decide to adopt MI, how clients experience MI-informed consultations, and how organisational factors shape post-training use. An example in veterinary MI research would be: what factors influence whether veterinary practitioners who have completed MI training (S) sustain MI skills in routine clinical practice after training (PI), explored through semi-structured interviews (D) using qualitative thematic analysis (R) to identify facilitators and barriers to ongoing use (E)?

For questions focused on evaluating services, programs, or implementation in real-world settings, SPICE provides a useful structure, specifying the Setting, Perspective, Intervention, Comparison, and Evaluation. SPICE is particularly well suited to implementation questions about whether and how MI can be embedded in routine veterinary practice across different organisational contexts. An example would be: in mixed veterinary practices in New Zealand (S), how do practice owners and clinicians experience the introduction of a structured MI training program (P), compared with no formal communication training (I/C), in terms of consultation quality, staff confidence, and client engagement (E)?

Before committing to a research question, applying the FINER criteria provides a practical final check. A good research question should be (1) Feasible given available time, resources, and access, (2) Interesting to the researcher and to the field, (3) Novel in that it adds something new, (4) Ethical in that it can be conducted with appropriate regard for participant welfare and consent, and (5) Relevant in that answering it will improve practice, animal welfare, or professional outcomes in a meaningful way. In veterinary MI research, feasibility deserves particular attention given the practical constraints of recording clinical consultations, recruiting sufficient numbers of practitioners, and sustaining follow-up across the timeframes needed to detect meaningful behaviour change.

## 5. Designing MI Research Studies

Designing robust MI research studies in veterinary clinical settings presents real practical challenges. Recording and coding clinical consultations to assess fidelity is currently labour-intensive, the behaviour changes and outcomes that MI aims to facilitate may take months to years to happen, and the sample sizes needed to detect meaningful effects have exceeded what most studies to date have been able to achieve. Where resource constraints limit what is possible, researchers need to be explicit about the implications for what can and cannot be concluded from their findings. This section addresses the key design decisions that determine whether a study will produce findings worth acting on, covering study design, outcome and fidelity measurement, sample size estimation, recruitment and retention, and ethical considerations specific to veterinary research contexts.

### 5.1. Choosing a Study Design

Selecting an appropriate study design is the first major methodological decision in any MI research project and should follow directly from the research question as well as the stage of the implementation pathway being investigated (Table 2).

Studies at the Exploring MI stage are primarily concerned with understanding the factors that influence whether practitioners engage with MI training and whether organisations support that engagement. Cross-sectional surveys are well suited to mapping awareness of MI across veterinary professional communities, identifying attitudes toward evidence-based communication practice, and characterising the organisational conditions that enable or prevent training uptake. Qualitative interviews and focus groups are particularly valuable for understanding the reasoning behind these decisions in depth, including the professional identity factors, workload pressures, and peer influences that shape whether a practitioner pursues MI training at all. Littlewood and Gardner provide detailed practical guidance on designing and conducting qualitative studies in veterinary contexts [32].

Training evaluation studies at the Learning MI stage are primarily concerned with whether practitioners can be trained to deliver MI with fidelity and what training formats and doses are most effective. Pre–post designs comparing practitioner communication behaviour before and after training are the most commonly used approach in the veterinary literature and are appropriate where a full RCT is not feasible. Where resources allow, RCTs or quasi-experimental designs comparing different training formats, doses, or delivery modes provide stronger evidence about what works and for whom. Longitudinal follow-up is essential for understanding whether skills are maintained after training ends, since studies that assess fidelity only immediately post-training tell us whether training worked but not whether skills persist in clinical practice over time, which is often the more important question.

Studies at the Delivering MI stage largely evaluate whether MI-consistent communication produces meaningful changes in client behaviour and animal welfare. This is where the evidence base is weakest in the veterinary literature and where the most methodologically demanding designs are required. Cluster RCTs, where practices or practitioners rather than individual clients are randomised, are the preferred design because they avoid the contamination problem that arises when the same practitioner delivers both MI and control conditions to different clients. Where cluster randomisation is not feasible, pragmatic trials comparing MI-trained practitioners with untrained controls, or pre–post designs with a matched comparator group, offer more achievable alternatives.

A further consideration in efficacy studies is that meaningful behaviour change in veterinary contexts may require more than a single consultation. Complex behaviour change targets such as chronic disease management, farm biosecurity, or long-term preventive care often involve revisiting ambivalence across multiple interactions over time, and a study design that treats MI as a single-session intervention may underestimate its potential effects. Where the clinical context reasonably involves multiple contacts, study designs should account for this by specifying the number and frequency of MI-informed consultations as part of the intervention protocol, and follow-up periods should be sufficient to capture behaviour change that accumulates across interactions rather than expecting measurable change from a single encounter.

Research at the Experiencing MI stage focuses on how clients perceive and respond to MI-informed consultations. Qualitative interviews and focus groups are the most appropriate designs for exploring client perceptions in depth, including how they experience the consultation, whether they feel heard and understood, and how the interaction influences their engagement with recommended changes. Mixed methods surveys combining structured scales with open-ended questions offer a more scalable alternative where broader reach is needed. A particular consideration in veterinary contexts is that client experience research needs to account for the triadic nature of the consultation, since clients are navigating their own emotional responses, their relationship with their animal, and their assessment of what is practically achievable, all within a single interaction.

Studies examining MI mechanisms in veterinary contexts are concerned with whether the processes through which MI produces behaviour change in human healthcare operate in the same way when the client is a proxy decision-maker acting on behalf of an animal. Sequential analysis of recorded consultations, examining the moment-to-moment relationship between practitioner MI-consistent behaviours and client change talk and sustain talk, is the primary design for this type of research. Where mechanism questions are embedded within a larger trial, mediation analysis can be used to test whether change talk or therapeutic alliance mediates the relationship between MI delivery and behaviour change outcomes. These designs require high-quality recordings of real consultations and trained coders applying validated instruments and are resource-intensive relative to other study types.

### 5.2. Measuring Fidelity

Without objective assessment of practitioner MI skills, it is impossible to interpret what a study’s findings mean, regardless of whether the focus is training evaluation, efficacy, or client experience. A training study showing no improvement in client outcomes cannot distinguish between MI being ineffective and MI not having been delivered properly, and the same interpretive problem applies across all study types.

The Motivational Interviewing Treatment Integrity code version 4.2.1 (MITI 4.2.1) is the most widely used and validated fidelity instrument and should be the default choice for studies requiring comprehensive assessment of practitioner MI skills [23,33]. The MITI evaluates practitioner behaviour across relational and technical domains, and core MI skills, and produces both global scores and behavioural counts that can be compared against established competency thresholds. The MITI has been revised over time based on research evidence about MI practice and mechanisms. A practical constraint is that the psychometric properties of the MITI were evaluated on a 20 min segment of behaviour change focused consultations. While the MITI has been applied in human behaviour change contexts involving shorter interactions, its psychometric properties in these settings have not been comprehensively evaluated. This presents a real challenge in veterinary practice where appointments are often considerably shorter and where behaviour change may be only one component of a longer clinical interaction covering multiple concerns. Where consultations are shorter, researchers should either design the study around a dedicated behaviour change interaction rather than a routine clinical appointment or select an alternative fidelity instrument that is validated for briefer encounters. Validating fidelity instruments for the specific demands of veterinary consultations remains an important research priority.

The Behaviour Change Counselling Index (BECCI) offers a validated alternative better suited to briefer consultations [34]. Developed by Lane and colleagues as a measure of practitioner competence in behaviour change counselling (an adaptation of MI designed for time-limited human healthcare settings), the BECCI uses a small number of globally rated items assessing behaviours such as inviting clients to talk about behaviour change, encouraging change talk, and conveying respect for client autonomy. The BECCI was designed to be scored from audio recordings with minimal coder training, making it more practical for routine use than the MITI, and has demonstrated acceptable validity, reliability, and responsiveness to training [34]. Its focus on the spirit and principles of MI rather than granular behavioural counts makes it particularly well suited to shorter consultations where there is insufficient consultation length to generate reliable MITI scores.

Several instruments assess client behaviour and experience alongside or instead of practitioner behaviour. The Motivational Interviewing Skill Code version 2.1 (MISC 2.1) is the most comprehensive fidelity instrument, coding both practitioner and client speech from audio-recordings across two passes providing detailed measurement of MI consistent and MI-inconsistent practitioner behaviour as well as behaviour counts, and measurement of change talk and sustain talk [35]. The MITI subsequently emerged from the MISC as a more focused instrument assessing practitioner behaviour only, substantially reducing coder training requirements and coding time. The Client Language Easy Rating (CLEAR) system is a simplified adaptation of MISC that codes client language only, categorising utterances as change talk or sustain talk in a single pass without requiring transcripts [36]. Unlike the MISC, the CLEAR does not code neutral client language, making it a more efficient tool for studies where the primary interest is the ratio of change talk to sustain talk rather than a comprehensive account of in-session client speech. The CLEAR is particularly relevant for mechanism and efficacy studies where client language is itself an outcome of interest. Together, the MITI and the CLEAR allow researchers to assess practitioner and client behaviour independently and with considerably less resource burden than the full MISC2.1 The Client Evaluation of Motivational Interviewing scale (CEMI) offers a further alternative, capturing client self-reported perceptions of the relational and technical dimensions of MI in a session using an 11-item measure completed immediately following the consultation [37]. The CEMI provides a low burden complement to observational coding, providing the client’s perception of the conversation, rather than observed MI fidelity.

Regardless of which instrument is used, reliable manual fidelity coding requires coders who have received formal training in the relevant instrument and can demonstrate acceptable inter-rater reliability before coding begins. Studies should report coder qualifications, training received, and inter-rater reliability statistics. Coding by a single researcher without reliability checks produces data that cannot be independently verified and should be avoided.

Fidelity assessment requires recordings of consultations, and obtaining these in real clinical settings introduces practical and ethical challenges that need to be addressed at the study design stage. Audio recording is generally sufficient for MITI and BECCI coding and is less intrusive than video for both practitioners and clients. Practitioners commonly report behaving differently when recorded, and this Hawthorne effect can artificially inflate fidelity scores relative to unobserved practice [38]. Strategies for reducing this include allowing a familiarisation period before study recordings begin, limiting the number of consultations recorded per practitioner, and framing recordings as a learning tool rather than an evaluation. Client consent procedures need to be clearly explained, and studies should report consent rates as a feasibility indicator, since low consent rates can introduce systematic bias by over-representing more engaged or trusting clients.

Artificial Intelligence (AI)-assisted coding tools for MI fidelity assessment are currently in development and have the potential to substantially reduce the time and training burden associated with manual coding [39,40]. Emerging platforms such as Lyssn (https://www.lyssn.io/resources/insights/predicting-mi-fidelity-like-a-human) (Accessed on 25 May 2026), draw on the MISC and MITI frameworks to generate automated measures of practitioner behaviour and client change talk and sustain talk, reducing both the time and error associated with manual coding. While these tools show promise, their reliability and validity in veterinary consultation contexts have not yet been established and should be evaluated before being adopted as a substitute for validated manual coding procedures.

### 5.3. Measuring Outcomes

Outcome selection should be driven by the research question and the stage of the implementation pathway being studied. Table 3 maps the key outcome domains relevant to MI research in veterinary contexts, the instruments available for measuring them, and the practical considerations that shape their use. A common weakness in the existing veterinary MI literature is the use of proximal outcomes such as self-reported confidence or knowledge as substitutes for the distal outcomes that actually matter, and researchers should be explicit about where in the causal chain their chosen outcomes sit and what assumptions are required to link them to meaningful change in veterinary practice.

Measuring client behaviour change presents challenges in veterinary contexts. Self-report is the most used approach but is subject to social desirability bias, with clients likely to over report adherence to recommendations when asked directly by the practitioner or a member of the practice team. Where possible, self-report should be supplemented with objective measures drawn from clinical records, direct observation, or species-specific clinical parameters. The timing of outcome assessment is also critical. Most existing veterinary MI studies assess outcomes at a single time point immediately after the consultation, which cannot distinguish meaningful long-term change from temporary short-term compliance, and behaviour change may simply not be detectable within the short follow-up periods that have characterised the field to date.

Welfare and production outcomes are the most meaningful but also the most difficult outcomes to study in veterinary MI research. They are inherently context and species-dependent, and researchers need to select clinically validated measures that are appropriate for the specific behaviour change target rather than relying on generic welfare indicators. A study examining MI in dental home care conversations should measure dental scoring while a study in dairy herd health management might measure somatic cell counts, lameness prevalence, or antibiotic use. The causal pathway from consultation through client behaviour change to welfare or production outcome needs to be explicitly justified in study design, and sufficient follow-up must be built in to allow downstream effects to become detectable. It is also worth noting that client behaviour change and animal welfare do not always move together. A client may make the recommended changes without producing measurable welfare improvement, either because the behaviour change was insufficient, because other factors are more influential, or because the follow-up period was too short. Treating behaviour change as a proxy for welfare improvement without testing that assumption directly is a limitation that should be acknowledged.

The CEMI is the only validated instrument specifically designed to measure client perceptions of MI-consistent consultation qualities, including perceived empathy, autonomy support, and collaboration [37]. It has not been validated in veterinary contexts, and its items were developed with human healthcare populations in mind, so direct application to veterinary settings requires caution. General client satisfaction scales are widely available but tend to produce high scores regardless of communication style, creating ceiling effects that limit their ability to detect differences between MI and standard consultation approaches. Researchers interested in client experience outcomes may need to supplement existing instruments with qualitative methods or develop veterinary-specific adaptations, and this is itself a worthwhile research priority.

### 5.4. Estimating Sample Size

Inadequate sample size is one of the most consistent methodological limitations in the veterinary MI literature. The quasi-experimental study by Svensson and colleagues examining herd health management outcomes calculated post hoc that 182 veterinarians would have been needed to detect meaningful differences, compared with the 36 who participated, illustrating how substantially underpowered studies in this field have been [41]. Sample size estimation should be conducted prospectively at the study design stage and the calculations as well as their assumptions should be reported transparently in published work.

Sample size requirements depend on three key decisions that researchers need to make explicitly before calculating. The first is the choice of primary outcome measure, since continuous outcomes such as MITI global scores or a validated adherence scale typically require smaller samples than dichotomous outcomes such as whether a client achieved a defined behaviour change target. The second is the minimum difference between groups that would be considered clinically meaningful, which should be determined by what would constitute a practically important change in the outcome rather than by what is statistically detectable with the available sample. The third is the expected variability in the outcome measure, which requires either an estimate from existing literature or data from a pilot study.

For studies where a single practitioner delivers MI to all participants, standard sample size calculations based on the number of clients are appropriate. This design eliminates between-practitioner variability as a source of confounding but produces findings with limited generalisability since results reflect that individual’s skill level and clinical context.

Multi-practitioner designs are almost always preferable on generalisability grounds but introduce clustering that standard sample size calculations do not account for. When clients are nested within practitioners, observations within the same practitioner are more similar to each other than observations across different practitioners, violating the assumption of independence that underlies standard statistical tests. The degree of clustering is quantified by the intra-class correlation coefficient (ICC), and even a modest ICC can substantially inflate the required sample size. Researchers should identify an appropriate ICC estimate from existing literature and apply a design effect correction to their sample size calculations. Practical guidance on clustered sample size estimation is available in standard biostatistics texts and through software tools including Stata, R, and G*Power, and statistical advice should be sought at the study design stage rather than after data collection.

For training evaluation studies, the practitioner is typically the unit of analysis rather than the client, and sample size calculations should be based on the number of practitioners rather than the number of consultations recorded per practitioner. Researchers also need to consider how many consultations per practitioner are required to obtain a reliable estimate of fidelity, since a single recorded consultation may not be representative of a practitioner’s typical performance. Researchers should be realistic about how many practitioners they can recruit, train, and retain over a longitudinal study period given the significant time investment participation requires. Where the evidence base is insufficient to support reliable effect size estimation, pilot and feasibility studies should be conducted before a full trial, with the goal of establishing recruitment rates, consent rates, recording feasibility, practitioner retention, and the variability of key outcome measures rather than testing effectiveness.

Attrition is a particular concern in longitudinal veterinary MI studies and should be anticipated in study size calculations at the design stage rather than managed retrospectively. MI-specific practitioner attrition data are limited, and veterinary-specific figures are essentially absent from the literature. A systematic review of knowledge translation studies targeting musculoskeletal rehabilitation practitioners found weighted enrolment, adherence, and retention rates of 82%, 74%, and 65% respectively, with rates declining substantially in longer studies and where participation was voluntary rather than employer-supported, which provides a useful benchmark for planning purposes [42].

### 5.5. Recruiting and Retaining Participants

Recruitment and retention present practical challenges in veterinary MI research because participation requires a significant and sustained time investment from practitioners who are already working in high-demand clinical environments. These challenges need to be anticipated at the study design stage and reported transparently in published work, since failure to do so obscures the degree to which study samples represent typical veterinary practitioners and clients. Table 4 summarises the most common recruitment and retention challenges in veterinary MI research, their consequences for study validity, and recommended mitigation strategies.

Several strategies have been shown to improve retention in healthcare practitioner research and are worth building into veterinary MI study designs from the outset. Systematic contact and scheduling, including regular reminders, clear timelines, and designated points of contact within the research team, are among the most consistently reported effective retention strategies across healthcare research [43]. Financial incentives, particularly cash rather than non-monetary alternatives, are associated with higher retention rates, though the level of incentive should be proportionate to the participation burden to avoid attracting unrepresentative samples [44]. Studies of shorter duration with fewer follow-up points tend to achieve higher retention [42], which creates a real tension with the longitudinal designs. Where long follow-up periods are unavoidable, minimising the burden of each individual data collection point can help sustain practitioner engagement over time. Where recruitment fell short of target or attrition was higher than anticipated, authors should discuss the implications for statistical power and the generalisability of findings.

### 5.6. Ethical Considerations

All veterinary MI research involving recorded consultations, client data, and animal welfare outcomes should be submitted for ethical review through the relevant institutional or national research ethics framework before data collection begins. The ethical dimensions of using MI in veterinary clinical practice, including the manipulation concern, the proxy motivation problem, practitioner competence, and the limits of collaborative practice when animal welfare is compromised, are addressed in detail in the companion narrative review [13]. This section focuses on the ethical considerations specific to conducting MI research in veterinary clinical settings. Table 5 summarises the key ethical issues, their implications for study validity or participant welfare, and recommended mitigation strategies.

Obtaining informed consent from both practitioners and clients requires careful attention to how participation is framed. Practitioners need to understand that their consultations will be recorded and assessed, and that fidelity coding will evaluate their communication behaviour against defined criteria. This creates a dual relationship where the researcher may simultaneously function as a trainer and an evaluator, and practitioners may feel implicitly pressured to participate or to perform differently when recorded. These dynamics should be acknowledged openly in participant information and managed through clear separation of research and training roles where possible.

Client consent in veterinary settings carries additional complexity because the consultation involves not only the client but also an animal whose welfare may be directly affected by the research. Clients must be able to decline participation without any risk to the care their animal receives, and this needs to be communicated unambiguously. Where clients are recruited through their usual veterinary practice, the pre-existing relationship with the practitioner may create subtle pressure to consent, and recruitment procedures should be designed to minimise this as far as possible. Client vulnerability also deserves explicit consideration, since consultations involving chronic disease management, end-of-life decisions, or significant welfare concerns may involve clients who are distressed, grieving, or under financial pressure. Researchers need clear protocols for managing situations where a client’s distress exceeds what the research context can appropriately contain.

Recordings of consultations contain identifiable personal and animal health information and must be managed with appropriate data security measures throughout the study. This includes encrypted storage, secure transmission, restricted access, clear data retention policies, and protocols for what happens to recordings at study end. Where recordings are shared with external coders, data sharing agreements should specify how confidentiality will be maintained and what happens to copies after coding is complete.

The animal welfare implications of outcome measurement deserve explicit consideration in ethics applications. Where welfare or production outcomes require physical data collection, such as blood sampling, body condition scoring, or handling for clinical assessment, the procedures involved require ethical justification in their own right, separate from the MI intervention itself. Researchers should ensure that any animal handling or sampling required for outcome measurement is minimised, appropriately justified, and conducted by trained personnel according to relevant animal welfare regulations.

## 6. Reporting MI Research Findings

Reporting the methodology and results from MI research in a consistent format is important for allowing comparison between different studies and settings. A recurring problem in the veterinary MI literature is that incomplete or inconsistent reporting makes it difficult to determine what was actually done, whether MI was delivered with sufficient fidelity to produce the intended effects, and whether the study was adequately powered to detect meaningful differences. Table 6 summarises the minimum reporting requirements for the methods section of a veterinary MI study, with MI-specific considerations called out alongside the standard elements expected in any clinical research publication. Adhering to these requirements does not guarantee a well-designed study, but it does ensure that readers have the information they need to evaluate the study’s strengths and limitations for themselves.

Describing the MI intervention and the training program used to deliver it in sufficient detail is one of the most important and most neglected reporting requirements in the MI literature. Without a clear account of what MI was delivered, who delivered it, how they were trained, and how fidelity was monitored, readers cannot determine whether the intervention constitutes MI at all, whether the training was adequate to produce proficient delivery, or whether the findings could be replicated in another setting. At a minimum, authors should specify the level of MI intended, whether full MI, adapted MI, or MI-informed communication, and describe how this was operationalised in practice. Training descriptions should include total contact hours, delivery format, whether practice with feedback on recorded consultations was included, and trainer qualifications. It is important to note whether fidelity assessment was conducted with simulated and/or real consultations since performance across these settings is not always correlated [45]. Where modifications were made to standard MI for the veterinary context, these should be described and justified rather than assumed to be self-evident.

The systematic reviews cited in the introduction to this paper found that poor intervention description and inadequate fidelity reporting were the primary factors limiting what could be concluded from the human healthcare MI literature [14,15,16,17]. While there has been a concerted effort among MI researchers and journal reviewers in recent years to improve reporting standards in human healthcare research, there is still gaps with reporting research studies in the smaller veterinary evidence base [13]. Consistent and transparent reporting is what allows individual studies to contribute to a cumulative evidence base, and the veterinary MI field has an opportunity to establish stronger reporting norms from the outset than have characterised the human healthcare literature.

## 7. Discussion

These guidelines provide a practical framework for designing, conducting, and reporting MI research in veterinary clinical settings, anchored by an implementation pathway that maps the journey from initial practitioner awareness through to sustained skilled delivery and meaningful outcomes for animals and their owners. Using this pathway as an organising framework for research is itself a novel contribution. Rather than treating MI as a single intervention to be tested against a control condition, it encourages researchers to identify precisely where in the pathway their work sits, what questions remain unanswered at that stage, and what study designs and outcome measures are appropriate for answering them. This approach has the potential to produce a more coherent and comprehensive evidence base than the field has generated to date.

The most significant practical challenge in veterinary MI research remains achieving and measuring fidelity in a clinical setting that looks very different from the addiction counselling and mental health contexts where MI was originally developed. Progress on this front will require methodological innovation alongside investment in sustained research programs, and the field will need to develop veterinary-specific adaptations of existing fidelity tools and outcome frameworks rather than relying on direct translation from human healthcare contexts. A mature veterinary MI evidence base would extend well beyond training outcomes to encompass the full pathway from organisational adoption through to sustained client behaviour change and measurable improvements in animal welfare, with studies that are adequately powered, longitudinally designed, and built around objective fidelity assessment in real clinical settings.

Beyond fidelity assessment, the field would benefit from research examining whether MI’s mechanisms operate differently under veterinary medicine’s proxy decision-making structure, where clients act on behalf of an animal who cannot participate in the conversation. This is difficult to study directly, since there is no naturally occurring comparison group of veterinary clients who are not acting as proxies. One option is to draw on existing paediatric and caregiver-mediated MI research as an external comparison, testing whether the same change talk to behaviour change associations hold when the beneficiary of change is not the person in the room. Another is within-sample comparison, contrasting consultations where the client stands to benefit personally from the outcome, such as zoonotic disease risk to the household, against consultations where the benefit is experienced only by the animal, to see whether MI’s mechanisms behave differently depending on who bears the cost and who receives the benefit.

Much of the guidance in this paper is drawn from human healthcare research because veterinary-specific evidence on fidelity assessment, training design, and outcome measurement remains limited. This reflects where the field currently sits rather than an assumption that human healthcare approaches will translate directly to veterinary contexts. Key questions that remain unanswered include whether MITI competency thresholds developed for longer dedicated behaviour change sessions are appropriate benchmarks in time-pressured veterinary consultations, whether the relationship between change talk and behaviour change holds when the client is acting as a proxy decision-maker for an animal, and what follow-up periods are actually needed to detect meaningful welfare outcomes. As the veterinary evidence base grows, these guidelines will need to be revisited and updated. Researchers are therefore encouraged to treat them as a starting point rather than a fixed framework.

## 8. Conclusions

MI has shown promise across many fields, but a stronger evidence base is still needed in veterinary medicine to answer questions about how it should be implemented and what it can realistically achieve for animal welfare. The framework and standards proposed in this paper are intended to help researchers think critically about what their research is targeting, design more robust studies, and build findings that are comparable across studies and settings. Progress will require more rigorous fidelity assessment, longer follow-up periods, and outcome measures that extend beyond proximal communication change to capture what MI-consistent practice actually achieves for clients and the animals in their care. Some aspects of MI research methodology will still need to be developed and refined specifically for veterinary contexts, since existing frameworks do not fully address the distinctive challenges the triadic relationship and proxy motivation problem create. Addressing these gaps will require sustained investment, but the field now has a clearer methodological foundation to build on and enough evidence to make a strong case for further investment. Veterinarians who communicate well with clients produce better outcomes for animals, and MI offers a structured and evidence-informed approach for doing so. Building that evidence base is ultimately about helping the people who care for animals have better conversations, make better decisions, and produce better outcomes for the animals in their care.

## Figures and Tables

**Table 1:** Overview of sample MI research questions in veterinary clinical settings organised by implementation pathway stage.

Pathway Stage	Key Research Questions
Exploring MI	What factors influence a veterinary practitioner’s decision to engage with MI training, including prior communication training, attitudes toward evidence-based practice, and perceived relevance to clinical work? What organisational conditions, including employer support, workload, and peer networks, enable or prevent practitioners from accessing MI training? How does awareness of MI spread within veterinary professional communities, and what sources of information are most influential?
Learning MI	Can veterinary practitioners be trained to deliver MI with fidelity using current training formats, and what training dose is sufficient to reach competency thresholds? What training formats, including workshops, online modules, and blended approaches, are most effective for building MI skills in veterinary contexts? How well does competency demonstrated in simulated consultations predict proficiency in real clinical practice? Does MI skill persist over time without ongoing coaching and feedback, and if not, what maintenance strategies are effective?
Delivering MI	Does MI produce behaviour change in veterinary clients compared to standard consultation approaches? What level of MI skill is required to produce meaningful changes in client behaviour, and how does this vary across consultation types? How do practitioners calibrate their use of MI across consultations of varying complexity, duration, and client readiness? What practitioner, client, and consultation factors moderate the effectiveness of MI delivery in real clinical practice? In what clinical contexts, presenting problems, and client situations is MI most appropriate, and how should practitioners identify when a different approach better serves the consultation? How do practitioners navigate the tension between respecting client autonomy and advocating for the welfare needs of an animal who cannot participate in the conversation?
Experiencing MI	How do veterinary clients experience consultations in which MI is used, and does this differ from their experience of standard consultations? How does exposure to MI-consistent communication affect client engagement, perceived empathy, and trust in the practitioner? How does client readiness to change, financial constraints, and the human–animal bond shape their responsiveness to MI within a consultation?
Outcomes	Does MI produce short-term changes in client behaviour, including adherence to treatment recommendations and follow-up attendance? Do behaviour changes produced through MI consultations persist over time, and what factors support or undermine maintenance? What are the downstream effects of sustained MI implementation on animal welfare and performance outcomes? Does MI training affect practitioner wellbeing, job satisfaction, and experience of clinical consultations over time? What are the practice-level impacts of implementing MI, including effects on consultation length, client retention, and financial performance? Where MI does not produce the expected behaviour change, to what extent is the outcome attributable to the intervention itself versus situational constraints on client behaviour such as financial limitations, competing demands, or lifestyle factors?
Mechanisms	Do the mechanisms through which MI produces behaviour change operate in the same way when clients are making decisions on behalf of an animal rather than for their own direct benefit?

**Table 2:** Recommended study designs organised by MI implementation pathway stage.

Pathway Stage	Recommended Study Designs
Exploring MI	Cross-sectional surveys; qualitative interviews or focus groups
Learning MI	Pre–post designs; Randomised Controlled Trials (RCTs) or quasi-experimental designs comparing training formats or doses; longitudinal fidelity assessment where feasible
Delivering MI	Cluster RCTs; pragmatic trials in real clinical settings; pre–post designs where randomisation is not feasible
Experiencing MI	Qualitative interviews or focus groups; mixed methods surveys
Mechanisms	Sequential analysis of recorded consultations; mediation analysis within RCT or trial data

**Table 3:** Outcome domains relevant to MI research in veterinary clinical settings, with corresponding measurement instruments and practical considerations for their use.

Outcome Domain	What Is Being Measured	Validated Instruments	Practical Considerations
Fidelity	Whether MI was delivered with sufficient skill to produce the intended effects	Motivational Interviewing Treatment Integrity code (MITI 4.2.1) for comprehensive assessment; Behaviour Change Counselling Index (BECCI) for briefer consultations; Client Language Easy Rating (CLEAR) system for client verbal responses	See Section 5.2; fidelity must be assessed in real consultations rather than role-play alone
Client change talk	Ratio of preparatory and mobilising change talk to sustain talk within consultations, as a proximal indicator of behaviour change	Motivational Interviewing Skill Code (MISC) for comprehensive coding; CLEAR for a more streamlined alternative	Requires recorded consultations and trained coders; higher ratios of change talk to sustain talk predict subsequent behaviour change in human healthcare but this relationship has not been confirmed in veterinary contexts
Client behaviour change	Whether clients make and sustain the recommended management changes following MI-informed consultations	Context-specific behavioural checklists; clinical records; follow-up structured interviews; direct observation where feasible	Self-report is subject to social desirability bias and should be supplemented with objective measures where possible; timing of assessment is critical since behaviour change may not be detectable at short follow-up; behaviour change does not guarantee improvements in welfare and production outcomes
Animal welfare and production outcomes	The downstream effects of client behaviour change on animal welfare and production performance	Context and species-specific clinical measures selected for the behaviour change target, for example dental scoring for dental home care studies or somatic cell counts for mastitis management studies	The most distal outcome domain and the hardest to attribute to MI specifically; requires sufficient follow-up to detect change; outcome selection must be justified by a plausible causal pathway from the consultation through client behaviour change to the measured welfare or production parameter
Client experience	How clients experience MI-informed consultations, including perceived empathy, autonomy support, and satisfaction	Client Evaluation of Motivational Interviewing (CEMI); general client satisfaction scales	CEMI is the only MI-specific client experience measure and has not been validated in veterinary contexts; general satisfaction scores tend to be high regardless of communication style, creating ceiling effects that limit sensitivity
Practitioner outcomes	Practitioner wellbeing, burnout, job satisfaction, and confidence in MI use	Maslach Burnout Inventory; Copenhagen Burnout Inventory; self-efficacy and confidence scales	No veterinary-specific validated tools exist; self-reported MI skill consistently overestimates objectively measured fidelity and should not be used as a proxy for competency
Practice-level outcomes	The operational and financial impacts of MI implementation	Consultation length measured by time-motion studies; follow-up appointment rates from practice management records; average client transaction value	Rarely studied in MI research; requires access to practice management data; confounded by many factors beyond communication style

**Table 4:** Recruitment and retention challenges in veterinary MI research, their consequences, and recommended mitigation strategies.

Challenge	Consequence	Mitigation
Practitioner self-selection bias	Volunteers are likely more motivated and communication-oriented than typical practitioners, inflating positive findings	Acknowledge explicitly in limitations; where possible recruit through mandatory continuing professional development (CPD) or institutional programs rather than open volunteering
Client consent bias	Clients with more positive practitioner relationships more likely to consent to recording, over-representing engaged clients	Report consent rates; compare demographics of consenting and non-consenting clients where possible
Contamination	Trained practitioners share MI skills with untrained colleagues, or deliver both MI and control conditions to different clients, obscuring true intervention effects	Use cluster randomisation; consider wait-list control designs; monitor for contamination through practitioner self-report
Differential attrition	Control group participants seek MI training independently once aware of it, or drop out at higher rates than intervention group, biasing results	Use wait list control designs where feasible; monitor and report dropout rates by condition; conduct intention-to-treat analysis
Practice-level gatekeeping	Practice owners or managers control staff participation, adding an organisational recruitment layer and potentially filtering out practices with less supportive cultures	Engage practice owners early in study design; consider practice-level incentives alongside practitioner-level incentives
Incentivisation effects	Financial or continuing professional development (CPD) incentives may attract different practitioner types than studies without incentives, affecting generalisability	Report incentives transparently; consider whether incentive level is proportionate to participation burden
Longitudinal attrition	Practitioners leave the study over time due to workload, job changes, or loss of interest, reducing statistical power and introducing bias	Minimise participation burden; maintain regular contact with participants; build attrition into sample size calculations

**Table 5:** Key ethical considerations in veterinary MI research, their implications, and recommended mitigation strategies.

Ethical Issue	Implication	Mitigation
Informed consent	Participants may not fully understand what participation involves, particularly regarding recording and fidelity assessment	Provide clear written information covering recording procedures, who will access recordings, how data will be used, and the right to withdraw without consequence
Client consent bias	Clients with more positive practitioner relationships more likely to consent, over-representing engaged clients and inflating positive findings	Recruit clients independently of the practitioner where possible; report consent rates and compare demographics of consenting and non-consenting clients
Practitioner coercion	Practitioners may feel implicitly pressured to participate, particularly where the researcher is also their trainer or employer	Ensure voluntary participation is explicitly stated; separate researcher and trainer roles where possible; allow withdrawal at any point without penalty
Dual relationship	Researcher functioning as both trainer and evaluator creates a power dynamic that may affect practitioner behaviour and study validity	Separate training and research roles where feasible; acknowledge the dual relationship explicitly in ethics applications and published limitations
Client vulnerability	Clients in distress around illness, end-of-life, or financial hardship may be emotionally vulnerable during recruitment and data collection	Establish clear protocols for recognising and responding to client distress; specify referral pathways; exclude actively distressed clients from recruitment where appropriate
Data privacy and security	Consultation recordings contain identifiable personal and animal health information	Use encrypted storage and transmission; restrict access to named researchers and coders; establish data retention and destruction policies; use data sharing agreements with external coders
Confidentiality	Practitioners and clients may be identifiable from published fidelity data or consultation descriptions	Anonymise all reported data; avoid describing consultations in sufficient detail to allow identification; obtain participant consent for any direct quotation
Hawthorne effect	Practitioners may alter their behaviour when recorded, inflating fidelity scores relative to unobserved practice	Allow a familiarisation recording period before study recordings begin; frame recordings as a learning tool; report potential Hawthorne effect as a limitation
Animal welfare	Control conditions may withhold communication approaches with potential to benefit animal welfare	Justify control condition design explicitly; consider wait list controls; specify welfare monitoring procedures during the study period
Third-party consent	Animals cannot consent to participation in research that may affect their welfare	Address explicitly in ethics applications; justify outcome measures in terms of animal welfare benefit; ensure welfare monitoring is built into study design

**Table 6:** Minimum reporting requirements for the methods section of veterinary MI research studies.

Reporting Element	What to Include	MI-Specific Considerations
Study design	Study design type, rationale for design choice, unit of randomisation or comparison	Justify why the chosen design is appropriate for the stage of the implementation pathway being studied; if cluster randomisation was not used, explain how contamination was managed
Setting	Clinical setting, practice type, country, consultation format	Describe species context, consultation length norms, and whether behaviour change was the primary purpose of the consultation or embedded within a broader clinical interaction
Practitioners	Number, recruitment method, eligibility criteria, relevant professional characteristics	Report prior communication training, years of experience, herd health or clinical caseload relevant to the study context, and any prior exposure to MI
Clients	Number, recruitment method, eligibility criteria, relevant demographic characteristics	Report human–animal bond context where relevant, financial constraints that may have affected participation or outcomes, and consent and dropout rates
Intervention description	What MI was delivered, by whom, in what format, over what duration	Specify the level of MI delivered using a defined framework; describe whether full MI, adapted MI, or MI-informed communication was intended and how this was operationalised; report any modifications made to standard MI for the veterinary context
Training program	Training content, format, duration, delivery method, trainer qualifications	Report total contact hours, whether training included role play or real consultation practice, whether MITI-coded feedback was provided, and trainer MI and MI training experience.
Fidelity assessment	Instrument used, coding procedure, who coded, when coding occurred	Report coder training, inter-rater reliability statistics, whether the coded consultations were from simulations or real client interactions, the minimum consultation length coded, and the proportion of consultations coded
Outcome measures	Primary and secondary outcomes, measurement instruments, timing of assessment	Specify whether outcomes are proximal, intermediate, or distal; justify the causal pathway from MI delivery to each outcome; report whether animal welfare or production outcomes were included and how they were measured
Sample size	Sample size calculation, effect size estimate, ICC if applicable, alpha and power levels	Report whether the practitioner or the client was the unit of analysis; report ICC estimate and source; justify the minimum clinically meaningful difference used in calculations
Statistical methods	Analytical approach, handling of clustering, missing data, intention to treat	Report how practitioner-level clustering was handled in the analysis; describe any adjustment for baseline differences between groups; report how missing data and dropout were managed

## Data Availability

No new data were created or analysed in this study. Data sharing is not applicable to this article.

## Abbreviations

The following abbreviations are used in this manuscript:

AI	Artificial Intelligence
BECCI	Behaviour Change Counselling Index
CEMI	Client Evaluation of Motivational Interviewing Scale
CLEAR	Client Language Easy Rating
CPD	Continuing Professional Development
FINER	Feasible, Interesting, Novel, Ethical, Relevant
ICC	Intra-class Correlation Coefficient
MI	Motivational Interviewing
MINT	Motivational Interviewing Network of Trainers
MISC	Motivational Interviewing Skills Code
MITI	Motivational Interviewing Treatment Integrity Code
OARS	Open-ended Questions, Affirmations, Reflections, Summaries
PACE	Partnership, Acceptance, Compassion, Empowerment
PICOT	Population, Intervention, Comparator, Outcome, Time
RCT	Randomised Controlled Trial
SPICE	Setting, Perspective, Intervention, Comparison, Evaluation
SPIDER	Sample, Phenomenon of Interest, Design, Evaluation, and Research Type
